# Possible Differences in Visual Attention to Faces in the Context of Mate Choice and Competition

**DOI:** 10.1007/s10508-025-03086-6

**Published:** 2025-02-25

**Authors:** Žaneta Pátková, Dominika Grygarová, Petr Adámek, Jitka Třebická Fialová, Jan Havlíček, Vít Třebický

**Affiliations:** 1 Faculty of Science, Charles University, Viničná 7, 128 44 Prague, Czech Republic; 2 Center for Advanced Studies of Brain and Consciousness, National Institute of Mental Health, Klecany, Czech Republic; 3 Third Faculty of Medicine, Charles University, Prague, Czech Republic; 4 Faculty of Physical Education and Sport, Charles University, Prague, Czech Republic

**Keywords:** Attractiveness, Formidability, Eye-tracking, Intrasexual selection, Intersexual selection

## Abstract

Existing research indicates that the shape of various facial regions is linked to perceived attractiveness and perceived formidability. Interestingly, little evidence shows that people directly focus on these specific facial regions during judgments of attractiveness and formidability, and there is little support for the notion that the levels of attractiveness and formidability affect raters’ visual attention. We employed eye-tracking to examine visual attention (the number of fixations and dwell time) in 40 women and 37 men, while they assessed 45 male faces in life-sized photographs for attractiveness and formidability. The facial photographs were grouped by varying levels of attractiveness and formidability (low, medium, and high). Our results showed that regardless of the characteristics rated, both men and women paid the most visual attention to the eyes, nose, mouth, and forehead regions. We found statistically discernible variation in visual attention in relation to the rater’s sex or target’s attractiveness levels for other facial features (the chin, cheeks, or ears), but these differences may not be substantial enough to have practical implications. We suggest that the eyes, the nose, and the mouth regions play a central role in the evolution of face perception as regions most salient to the acquisition of informative cues about others. Further, during both attractiveness and formidability judgments, men looked longer at the stimuli than women did, which may hint at increased difficulty of this task for men, possibly because they compare themselves with the stimuli. Additionally, irrespective of sex, raters looked marginally longer at faces with a medium level of formidability than at those with a high formidability level, which may reflect ambiguity of these stimuli and uncertainty regarding assessment. We found no other significantly relationships between the target’s attractiveness and formidability levels and the rater's visual attention to whole faces.

## Introduction

People tend to spontaneously assess others for various characteristics, including those relevant to mate choice or conflicts (Little, 2014; Třebický et al., 2019). Based on these assessments, individuals can evaluate the suitability of potential partners or formidability of rivals and make appropriate decisions (Sell et al., 2012; Thornhill & Gangestad, 1999). These assessments are often based on visual cues, and the face is a particularly salient source of information (Calder et al., 2011).

It has been proposed that inter- and intrasexual selection jointly drive sexual dimorphism of human faces (Puts, 2010; Třebický & Havlíček, 2021; Třebický et al., 2012). Intersexual selection influences facial traits which are considered attractive and believed to provide cues to an individual’s mating quality, such as health or immunocompetence (Stephen & Luoto, 2023 but see Jones et al., 2021; Pátková et al., 2022). Mating with individuals who have attractive traits can have both direct (e.g., parental care, access to resources) and indirect benefits (genetic material for an offspring), and thus increase an individual’s reproductive success (Kirkpatrick & Ryan, 1991; Little, 2014). Intrasexual selection, on the other hand, has probably shaped the development of morphological traits associated with success in confrontations, i.e., traits linked to perceived and actual formidability and dominance (Puts, 2010; Třebický et al., 2012). Formidability assessments are crucial for deciding whom to recruit as an ally and whether to engage in or avoid a physical conflict (Třebický et al., 2021). In other words, they help in deciding whether one stands a good chance of winning (and gaining access to mates and resources) or if it is preferable to avoid confrontation and the risk of defeat (and associated injuries).

Given the higher prevalence of physical aggression among men, intrasexual selection has been studied more frequently in men (Archer, 2004), but formidability assessments of men are relevant to women as well. For instance, the ability to assess formidable men can help both men and women to stay out of harm’s way. Formidability can also serve as a cue of mate quality: a formidable partner may provide advantages, including protection of offspring or heritable traits of formidability (Třebický et al., 2012). This choice, however, also carries potential costs: For instance, research suggests that formidable men need not be better at providing parental care or resources and that their aggression can be directed at their partners (Qvarnström & Forsgren, 1998; Snyder et al., 2008). Both men and women thus have good reasons to pay attention to visual cues of formidability and attractiveness in men. For women, both traits may primarily provide cues to the quality of potential mates, whereas for men, they can help assess the quality of potential rivals.

Multiple studies have investigated the role of individual facial features in attractiveness and formidability judgments. They identified several facial regions—such as the eyes, nose, mouth, chin, or jaw—whose shape (e.g., wider mouths and fuller lips, more angular jaws, thicker eyebrows) is in men associated with a higher perceived attractiveness (e.g., Windhager et al., 2011) and formidability (e.g., a broader chin, bigger nose and mouth, deep-set eyes, prominent eyebrows) (Třebický et al., 2013). Further, it has been shown that the shape of certain features (e.g., smaller eyes, shorter nose, or wider and more prominent lower jaw) is linked to a higher perceived facial masculinity and dominance (Windhager et al., 2011). However, even though the shape of certain (and multiple) facial regions may be associated with the rating of perceived characteristics, their perceptual importance can vary. Morphological growths of facial regions are interconnected (Enlow & Hans, 1996), so the shape of one area affects the shape of surrounding regions. For instance, the shape of the masseter muscle influences the shape of the mandible and zygomatic arch and, thus, the shape of the cheeks. Therefore, while the morphometrics-based perception studies find associations between various facial features and judgments of socially relevant characteristics, only some of those features might affect perception (and ratings), while others can be merely shape correlates. Another approach to inspecting the role of individual facial features in judgments, besides morphometrics and perception studies, is to assess visual attention.

Research frequently uses eye-tracking to investigate visual attention directly and to avoid potential biases associated with self-reports. Eye-tracking enables the collection of multiple measures of visual attention to areas of interest (AOI) within a visual stimulus, such as the number of fixations, i.e., the number of times the gaze rests at a particular location or AOI, and dwell time in the AOI, which sums the time spent looking at a particular AOI. A higher number of fixations and longer dwell time on a stimulus are interpreted as indicative of its importance, informativeness, likability, and possibly also of the associated cognitive load (Duchowski, 2017; Skaramagkas et al., 2023). Therefore, eye-tracking could help to disentangle the relevance of individual facial features to judgments of attractiveness and formidability.

Previous eye-tracking studies have provided some insights into visual attention to others. They showed that when presented with full-body photographs, both men and women direct their visual attention primarily to faces (Hewig et al., 2008). When visually exploring a face without a particular task, the eyes, the nose, and the mouth attract the most visual attention (Hickman et al., 2010; Król & Król, 2019; Semmelmann & Weigelt, 2018). Fewer studies have focused on visual attention to faces and their features within specific contexts. Zhang et al. (2017) reported that during attractiveness ratings of women’s faces, men and women looked the most at the nose, eyes, and lips. However, these were the only AOIs specified, which prevented obtaining information about how visual attention toward other features is allocated. Kwart et al. (2012) explored more AOIs and focused on differences in visual attention to faces and their features during ratings of age and attractiveness. They, too, found that in both tasks the majority of visual attention was directed toward the eyes and the nose. Similarly, Hermens et al. (2018) reported that during judgments of trustworthiness and dominance, visual attention is similar in both tasks, with the eyes, the nose, and the mouth attracting the gaze the most. On top of that, it has been demonstrated that more attractive faces and their features (the nose and the mouth, Kwart et al., 2012) are looked at longer and more often, and that this effect is probably stronger in assessments of faces of the preferred sex (Leder et al., 2016; Mitrovic et al., 2018; Valuch et al., 2015).

These findings contrast with the results of morphometric studies, which identified variation in other facial features, especially the jaw and the chin, as affecting both attractiveness and formidability judgments. Still, evidence regarding direct visual attention to particular facial areas in attractiveness and formidability judgments remains equivocal. Eye-tracking studies tend to employ a limited number of AOIs and rarely investigate visual attention during facial attractiveness rating and even less so during formidability judgments. Moreover, no eye-tracking studies have investigated the impact of different levels of perceived facial formidability on visual attention.

Some eye-tracking studies focus on the rating of bodies (e.g., Dixson et al., 2014; Durkee et al., 2018; Garza & Byrd-Craven, 2021; Pazhoohi et al., 2019), which can be used to draw parallels, especially in the case of formidability. Durkee et al. (2018) showed, using eye-tracking, that people pay attention to the chest and shoulders when assessing formidability, emphasizing the importance of upper-body musculature. These findings support the idea that upper-body strength serves as a predictor of formidability and has been shaped by intrasexual selection pressures (Puts, 2010). This finding was later supported by Pazhoohi et al. (2019), showing men looked longer at the chest region of men with higher (a cue to masculinity, strength and formidability) vs lower shoulder-to-hip ratio. Therefore, using eye-tracking, the studies were able to lay further evidence for the suggested importance of specific regions in a specific rating. Employing eye-tracking during the facial formidability and attractiveness rating should help us understand the processes underlying visual attention, which may be differentially directed to certain facial features significant in the evolution of human mate choice and competition.

In the present study, we use the findings of morphometric and perception studies to build predictions for an investigation using an eye-tracking approach. We have employed eye-tracking to examine men’s and women’s visual attention (the number of fixations and dwell time) to male faces and particular facial features during assessments of facial attractiveness and formidability. We have also investigated whether and how raters’ visual attention differs depending on the level (low, medium, and high) of targets’ facial attractiveness and formidability. To do so, we used life-sized high-resolution facial photographs with multiple AOIs. In addition to the eyes, the nose, and the mouth, we specified also other areas which research had shown to be relevant to facial attractiveness and formidability judgments, such as the cheeks and chin. We predicted that men’s and women’s visual attention to whole faces would increase (more fixations, longer dwell time) with increasing levels of targets’ facial attractiveness and formidability. Further, we predicted that majority of visual attention would be directed to the eyes, the nose, and the mouth, but other facial regions would also attract visual attention. Finally, we have explored possible differences in visual attention to whole faces and particular facial features in relation to the raters’ sex and targets’ levels of facial attractiveness and formidability.

## Method

The study was preregistered prior to data analysis (https://osf.io/5fnc2). Before enrolling in the study, all participants were informed about its goals and signed an informed consent form. Data used in this study are part of a larger project focused on neural and attentional processes in the visual perception of male faces and bodies using functional magnetic resonance imaging (fMRI) (Třebický et al., 2024a) and eye-tracking. Data collection took place in Q3–Q4 2019.

### Participants

We recruited participant raters for eye-tracking sessions via social media (Facebook), oral invitations, and posters in the halls of the National Institute of Mental Health, the Faculty of Science, Faculty of Humanities, Faculty of Physical Education and Sport, and student dormitories (all Charles University, Prague, Czechia). Requirements for participation were the following: being a healthy man or woman aged 18–40 years, having normal or corrected-to-normal vision (up to ± 5 diopters), not being pregnant, being right-handed, and without a history of seizures, significant head trauma, mental retardation, claustrophobia, or any other MRI contraindication (such as having metallic or electronic implants; Spaniel et al., 2016).

In total, 40 women and 40 men participated in the study but three of the men were above 40 years of age and therefore removed from further analyses. The resulting sample of raters thus consisted of 40 women (M = 24.9 ys, SD = 5.45, age range = 18–40) and 37 men (M = 24.8 ys, SD = 4.15, age range = 19–38). Age did not statistically discernibly differ between the sexes. For details, see Table S1 in Supplementary Materials.

### Measures and Procedure

As stimuli, we used 45 standardized facial photographs of men (M = 26.6 ys, SD = 5.86, age range = 18–38) obtained in previous studies (Třebický et al., 2018, 2019). All photographs were post-processed in Adobe Lightroom CS6 and Adobe Photoshop CC 2017 software for standardization of position (while preserving relative differences in head size between individuals), color, and exposure. The facial photographs were then exported in 1:1 scale into 8-bit JPEG format (3840 × 2160, 163 PPI, sRGB color space). For more details, see Třebický et al. (2018).

The data collection consisted of two consecutive sessions. In the first session, participants underwent an fMRI scanning session during which they rated the attractiveness and formidability of photographs of 45 male faces and 45 male bodies. This was followed by an eye-tracking session during which they likewise rated photographs of 45 male faces and 45 male bodies on 7-point scales for attractiveness and formidability. In both cases, the stimuli were rated for formidability and attractiveness consecutively, whereby the order of these two large blocks was randomized. Further randomization was then applied within the blocks (for details, see Sect. “Stimuli Blocks.”). Finally, participants were asked to complete a brief survey regarding their basic demographic data, such as age and sex. After the session, all participants received a debriefing leaflet with a detailed description of the study and 400 CZK (app. 16 EUR) in compensation for their time. The whole session lasted approximately 2 h. Only data from eye-tracking of facial photographs of the faces are considered in this paper.

#### Stimuli Reference Rating

The stimuli were rated for attractiveness and formidability in previous studies on 7-point scales (e.g., 1—not attractive, 7—very attractive) (Třebický et al., 2018). Based on these ratings, we created categories of faces with low (attractiveness rating: M = 2.17, SD = 0.30; formidability rating: M = 2.80, SD = 0.27), medium (attractiveness rating: M = 3.02, SD = 0.26; formidability rating: M = 3.80, SD = 0.33), and high (attractiveness rating: M = 4.00, SD = 0.35; formidability rating: M = 4.90, SD = 0.50) level of attractiveness and formidability. Each category consisted of 15 faces.

#### Stimuli Blocks

The design of stimuli presentation was analogous in the eye-tracking and the fMRI sessions to enable possible future comparisons and ensure complementarity of the studies. Stimuli images were presented in blocks. Aside from facial and body photographs, participants were also presented with a set of shuffled images in the form of mosaic pixelated stimuli images created by overlaying individual stimuli images from each stimulation set (five images per set, 45 sets in total). These were not rated: they functioned as a resting condition (for further details, see https://osf.io/u48tz).

**Fig. 1:**
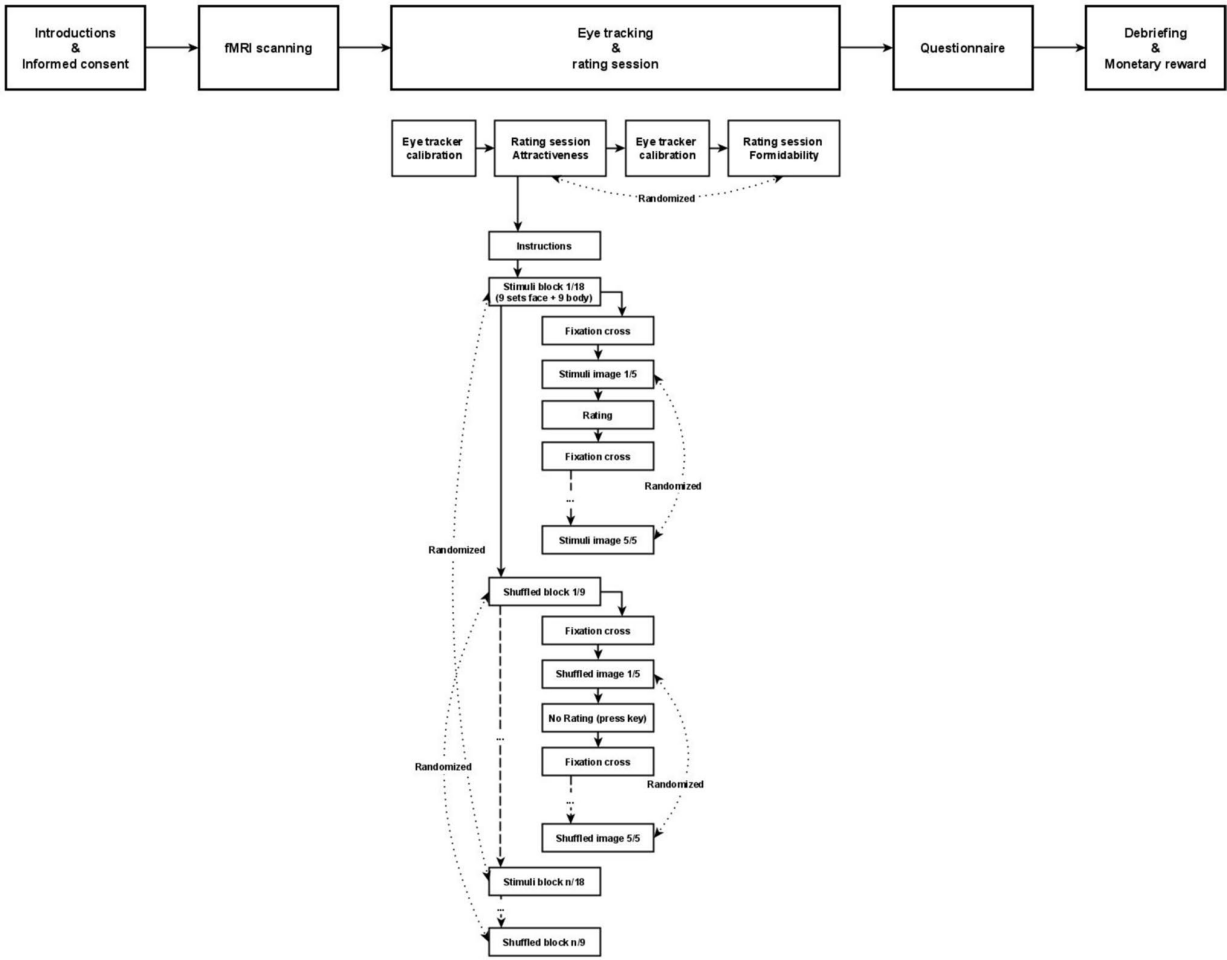
Procedure flowchart

Participants were thus presented with blocks of stimuli which varied in salience (high, medium, low) and with resting non-stimuli (the shuffled images) (Clark et al., 1998). Each rater was presented with 135 images in 27 randomized blocks (9 blocks of faces, 9 blocks of bodies, and 9 blocks of shuffled images) each of which contained five images in a randomized order; cf. Fig. 1.

#### Eye-Tracking

**Fig. 2:**
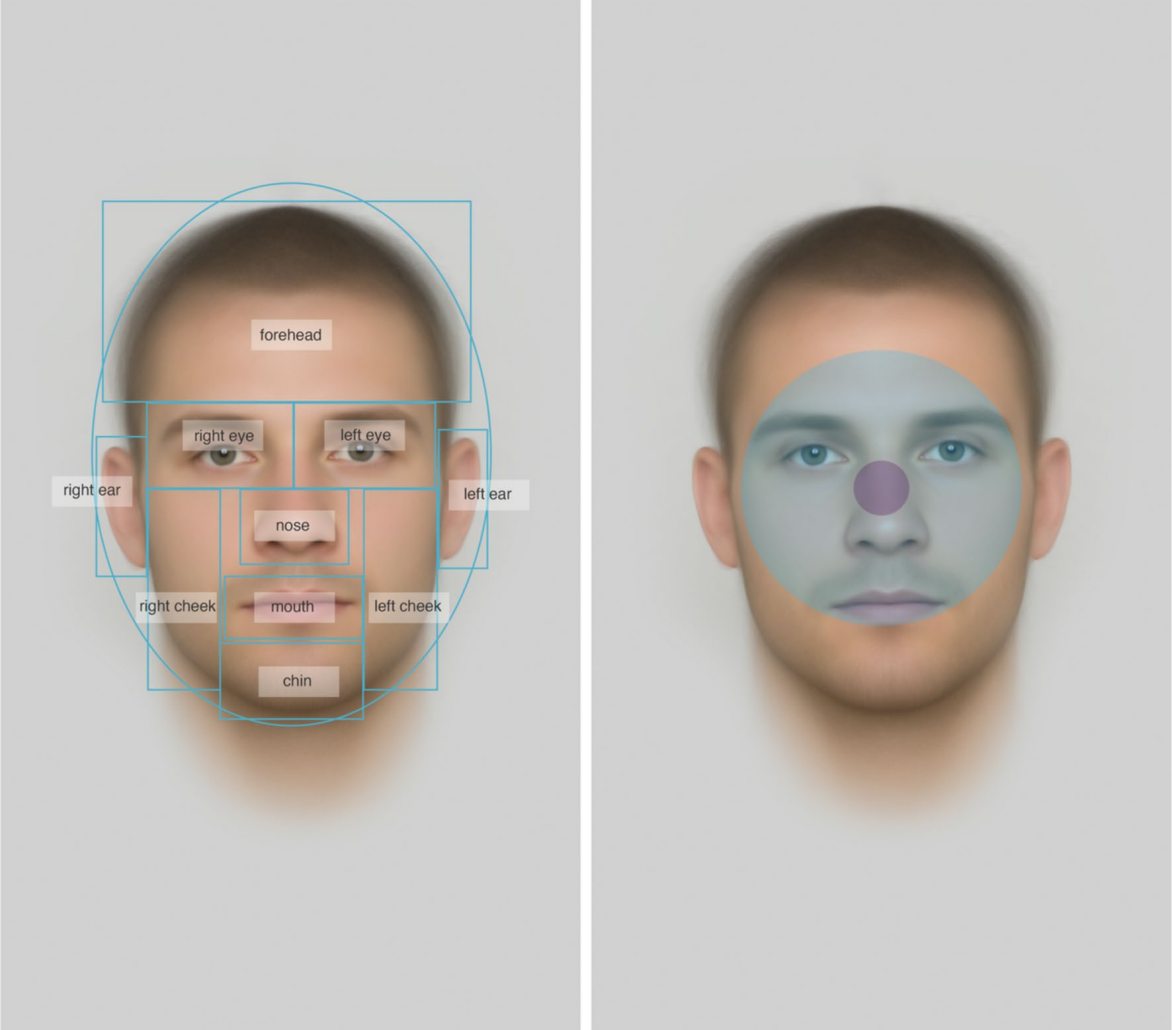
An example of delineated AOIs on the left (the ellipse indicates the “whole face” AOI) and visualization of the central vision on the right. The violet color represents an estimate of foveal vision, the blue color parafoveal vision, which jointly amount to central vision

The eye-tracking session took place in a quiet and windowless eye-tracking laboratory at the National Institute of Mental Health under standardized conditions (with artificial lighting). First, we determined the dominant eye of each participant using a variation of the Porta test (Crovitz & Zener, 1962). During the rating, we then tracked the dominant eye. Raters were seated in an office chair and rested their head on a head-and-chin rest (app. 109 cm from the screen, SR Research Head Support) to minimize any movement or change of position. We performed an eye-tracking calibration and validation using a 9-point calibration scheme (Blais et al., 2008). The rating session started only after calibration was successfully validated. The 109 cm distance from the screen and 4 K resolution results in areas of foveal (center of the field of vision with the highest visual acuity, ~ 2°angle of view) and parafoveal (area surrounding fovea with lesser vision acuity; ~ 10°angle of view) vision (Ivančić Valenko et al., 2020) covering 2% and 52% (40,454 px and 1,016,288 px) of the average stimulus face area, respectively (Fig. 2).

The rating task was created and conducted using the Experiment Builder (SR Research) on a 4 K 27″ LCD screen (Benq IPS; 3840 × 2160, 163 PPI, 99% sRGB color space coverage) pivoted into portrait orientation to accommodate life-sized facial photographs. The LCD screen was color- and luminance-calibrated using an X-rite i1 Display probe (connected during the experiment). Eye movements were recorded by an EyeLink 1000 Plus eye-tracker (SR Research Ltd. Ottawa, Ontario, Canada) (1000 Hz) and data collected by a host PC running Romdos 7.1 OS.

During the eye-tracking session, participants were instructed to rate the facial photographs using the 7-point scales twice: once for attractiveness (“How attractive do you find the man in the photograph?”; 1–least attractive, 7–most attractive) and once for formidability (“How successful would be this man in a physical encounter confrontation”?; 1–least successful, 7–most successful). The order of the tasks (attractiveness/formidability rating) was randomized.

Participants were first instructed to look at a fixation cross displayed for 1,000 ms in different quadrants of the screen (but not in the center of the screen to avoid fixations bias for the area where the stimuli were about to be presented). This was followed by a 4,000 ms-long presentation of facial photograph. On the following screen, participants were shown a 7-point verbally anchored scale of attractiveness/formidability, which they used to indicate their rating by clicking a mouse.

#### Delineation of AOIs

We have manually defined an array of AOIs for each stimulus using the Data Viewer software (4.3.1). In addition to the typically used eyes, nose, and mouth, we have also included features identified as potentially important for judgments of attractiveness and formidability by previous studies (Cunningham et al., 1990; Třebický et al., 2013; Windhager et al., 2011). The final set of AOIs thus included the whole face, the right eye, the left eye, nose, mouth, forehead (including hair), chin, the right cheek, the left cheek, the right ear, and the left ear. This set is similar to one used by Chelnokova and Laeng (2011). See Fig. 2 for individual AOIs; for AOI areas in px, see Table S2 in Supplementary Materials.

### Data Analysis

All statistical analyses were performed in jamovi (v 2.3.28) (The Jamovi Project, 2023). As in previous studies (e.g., Millen & Hancock, 2019), we considered only fixations ≥ 80 ms in the analyses. Aside from that, we noticed that for three raters, no fixations were recorded at some point during attractiveness rating. For the first rater, this occurred for five targets; for the second rater, it was the case for 20 targets. For the third rater, it occurred for one target. In the formidability rating, this happened for a combination of one rater and one target. All these cases were likely the result of eye-tracking signal loss, which is why we have excluded these trials from subsequent analyses (Leder et al., 2016; Rudolfová et al., 2022).

Visual inspection of the data distribution and the Kolmogorov–Smirnov test indicated that the data for the number of fixations and dwell time did not follow a normal distribution but a negative binomial. Therefore, we employed generalized mixed-effects models (GLMMs) using the GAMLj module (v 2.6.6) in jamovi.

The contexts (attractiveness/formidability ratings) were analyzed in separate models; visual attention to the whole face and to the individual AOIs was likewise analyzed separately. In separate models, the number of fixations and dwell time (ms) were entered as dependent variables.

The target’s level of attractiveness/formidability (high, medium, low) and the rater’s sex were entered as predictors for whole face analyses. For example, the relationship between the rater’s number of fixations on the whole face (a dependent variable), rater’s sex, target’s level of facial attractiveness (both predictors), and their interaction during facial attractiveness rating were assessed using the following model: N fixations on face ~ 1 + target’s attractiveness level + rater’s sex + target’s attractiveness level: rater’s sex + (1|ID rater). For formidability ratings, we used an analogous model. Subsequently, we ran models that included dwell time on whole faces. To control for the variability of targets and raters, we have first entered the rater’s and the target’s ID as random effects. Those models, however, exhibited a singular fit, which was due to virtually no variance of ID target and generally had a lower Akaike information criterion (AIC) when only the rater ID random effect term was included. Therefore, we report all analyses without the target ID random effect. For details, see https://osf.io/5fnc2.

For analyses of individual AOIs, we have entered as predictors the target’s levels of attractiveness/formidability, the rater’s sex, and the AOI. Based on the AIC or because the models did not converge when the ID target was included, we include only the ID rater as a random effect in the model. For details, see https://osf.io/5fnc2.

In a minor divergence from preregistration, we report *χ*^2^ and *p*-value for fixed-effect omnibus test results in the main text for the GLMMs, while fixed-effect parameter estimates using (exp)B with 95% CI for each model can be found in the Supplementary Materials. We used a post hoc test with Holm correction to test differences between pairs of predictor levels. Full results and observed power (Table S3) are reported in the Supplementary Materials.

We have set *p* ≤ 0.05 as a threshold for statistical discernibility. By using the term “statistically discernible” instead of traditionally used “statistical significance,” we want to indicate that the statistical test has found some evidence of a discernible effect but avoid any implication of its significance.

## Results

### Whole Face Analyses

#### Attractiveness

**Fig. 3:**
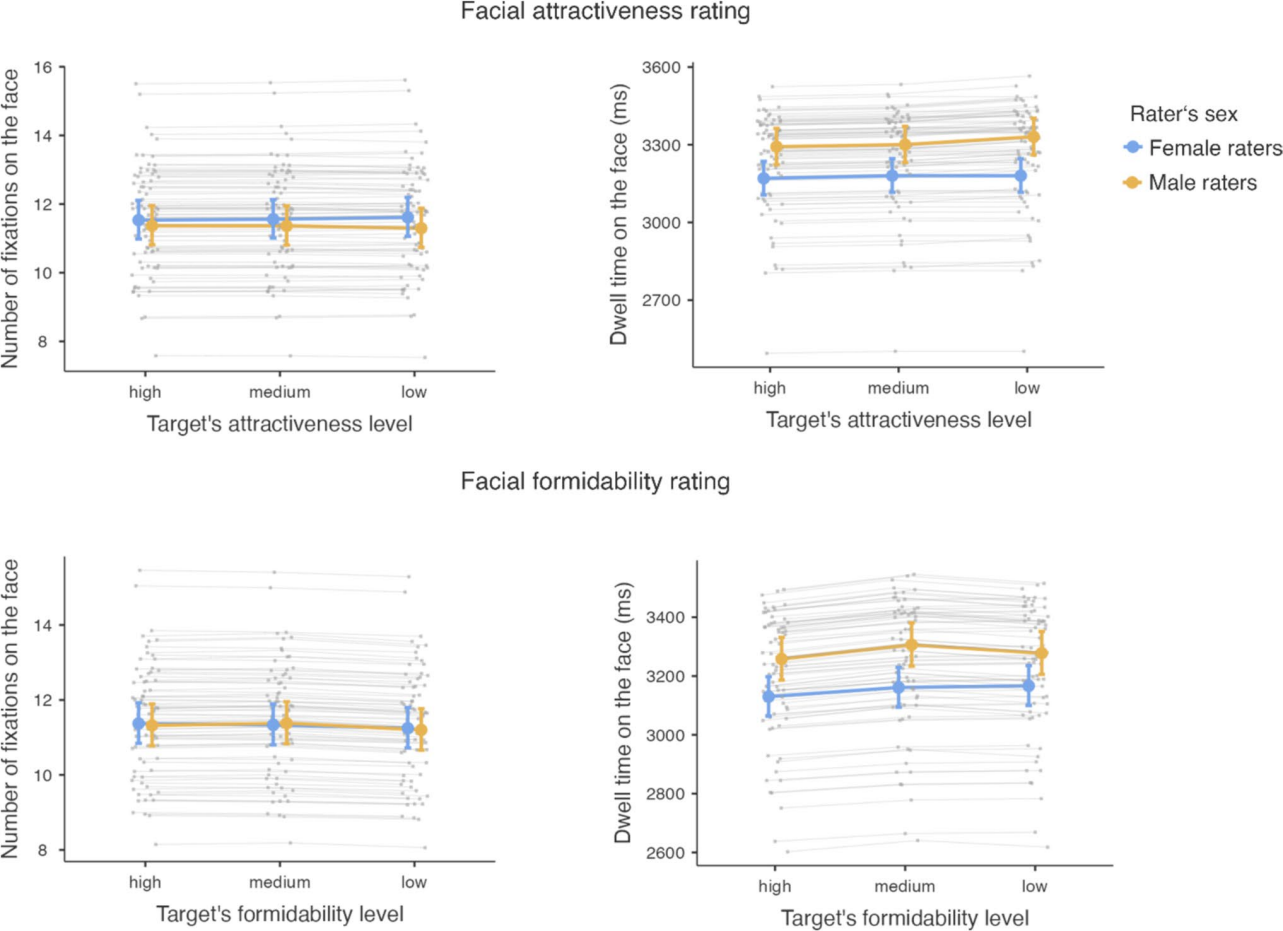
Differences in visual attention in relation to the target’s facial attractiveness level during facial attractiveness rating (top row) and in relation to the target’s facial formidability level during facial formidability assessment (bottom row), with the number of fixations on the whole face on the left and dwell time on the whole face on the right side. Colored dots represent mean values, error bars their 95% confidence intervals, blue color represents female raters, and yellow color male raters. Gray lines and dots represent random effects plotted by the ID rater

A GLMM showed that during the facial attractiveness rating task, the number of fixations on the whole face was not statistically discernibly predicted by the target’s level of facial attractiveness (*χ*^2^(2) = 0.005, *p* = 0.997), the rater’s sex (*χ*^2^(1) = 0.360, *p* = 0.549), or by an interaction of the two (*χ*^2^(2) = 0.371, *p* = 0.831) (Fig. 3).

A further GLMM showed that during the facial formidability rating task, the dwell time on the whole face was not statistically discernibly predicted by the target’s level of facial attractiveness (*χ*^2^(2) = 3, *p* = 0.223). It was, however, predicted by the rater’s sex (*χ*^2^(1) = 8.08, *p* = 0.004) but it wasn’t predicted by the interaction between the target’s level of attractiveness and rater’s sex (*χ*^2^(2) = 1.39, *p* = 0.498). Men spent statistically discernibly more time looking at whole faces than women did (3,308 ms vs 3,177 ms) when assessing male facial attractiveness (Table 1 and Fig. 3).

**Table 1:** Number of fixations and dwell time in AOIs for each context (attractiveness and formidability) and sex

Measure	AOI	Attractiveness		Formidability	
		Female raters	Male raters	Female raters	Male raters
		Mean with 95% CI [LL, UL]		Mean with 95% CI [LL, UL]	
Number of fixations	Whole face	11.6 [11.1, 12.1]	11.3 [10.8, 11.9]	11.3 [10.8, 11.8]	11.3 [10.8, 11.8]
	Right eye	3.32 [3.14, 3.50]	3.01 [2.84, 3.18]	3.26 [3.09, 3.43]	3.07 [2.91, 3.25]
	Left eye	3.43 [3.25, 3.62]	3.33 [3.15, 3.52]	3.29 [3.12, 3.47]	3.39 [3.21, 3.58]
	Nose	1.86 [1.76, 1.98]	1.82 [1.71, 1.93]	1.90 [1.80, 2.01]	1.73 [1.63, 1.83]
	Mouth	1.12 [1.05, 1.19]	1.01 [0.94, 1.08]	0.97 [0.91, 1.03]	0.88 [0.82, 0.94]
	Forehead	0.84 [0.78, 0.90]	1.14 [1.06, 1.22]	0.83 [0.77, 0.89]	1.00 [0.94, 1.07]
	Chin	0.09 [0.08, 0.11]	0.14 [0.13, 0.17]	0.12 [0.10, 0.14]	0.19 [0.16, 0.21]
	Right cheek	0.09 [0.07, 0.10]	0.07 [0.06, 0.08]	0.08 [0.07, 0.10]	0.12 [0.10, 0.14]
	Left cheek	0.07 [0.06, 0.08]	0.09 [0.07, 0.10]	0.06 [0.05, 0.08]	0.12 [0.10, 0.14]
	Right ear	0.06 [0.05, 0.07]	0.05 [0.04, 0.07]	0.10 [0.08, 0.12]	0.08 [0.07, 0.10]
	Left ear	0.08 [0.07, 0.10]	0.12 [0.11, 0.14]	0.11 [0.10, 0.13]	0.13 [0.11, 0.15]
Dwell time (ms)	Whole face	3177 [3116, 3239]	3308 [3242, 3375]	3153 [3089, 3218]	3281 [3212, 3351]
	Right eye	1030.5 [848.47, 1251.5]	957.9 [783.72, 1170.7]	1065.81 [853.84, 1330.4]	1023 [812.87, 1287.4]
	Left eye	1150.7 [944.86, 1401.3]	1129.1 [920.45, 1385.1]	1241.59 [993.06, 1552.3]	1177.11 [934.99, 1481.9]
	Nose	486.8 [399.42, 593.2]	498.1 [405.7, 611.5]	571.89 [457.55, 714.8]	471.04 [374.22, 592.9]
	Mouth	325.5 [267.24, 396.6]	280.6 [228.87, 344]	307.55 [246.12, 384.3]	261.86 [208.04, 329.6]
	Forehead	208.5 [171.2, 254]	325.4 [265.27, 399.3]	212.92 [170.68, 265.6]	286.1 [227.33, 360.1]
	Chin	20.4 [16.72, 25]	37.8 [30.78, 46.5]	27.26 [21.77, 34.1]	45.87 [36.39, 57.8]
	Right cheek	14.7 [11.92, 18]	15.8 [12.86, 19.5]	13.72 [10.96, 17.2]	26.24 [20.79, 33.1]
	Left cheek	13.9 [11.32, 17]	20.8 [16.98, 25.6]	14.14 [11.3, 17.7]	26.17 [20.76, 33]
	Right ear	10.5 [8.54, 12.9]	10.3 [8.39, 12.8]	9.98 [7.89, 12.6]	16.05 [12.7, 20.3]
	Left ear	18.5 [15.11, 22.7]	30.8 [25.03, 37.8]	18.76 [14.92, 23.6]	30.41 [24.12, 38.3]

Mean values are calculated based on the estimated marginal means of each respective model

#### Formidability

A GLMM showed that during the facial formidability rating task, the number of fixations on the whole face was not statistically discernibly predicted by the target’s level of facial formidability (*χ*^2^(2) = 1.128, *p* = 0.569), rater’s sex (*χ*^2^(1) = 0.002, *p* = 0.962), or by the interaction between the two (*χ*^2^(2) = 0.151, *p* = 0.927) (Fig. 3). For the *dwell time* during facial formidability rating of the whole face, the model showed that it was statistically discernibly predicted by the target’s formidability level (*χ*^2^(2) = 9.84, *p* = 0.007) and by rater’s sex (*χ*^2^(1) = 6.99, *p* = 0.008) (Fig. 3), but there was no statistically discernible interaction between the two (*χ*^2^(2) = 1.67, *p* = 0.435). During facial formidability assessment, both men and women exhibited statistically discernibly but just marginally longer dwell time on men’s faces with a medium rather than high level of facial formidability (3,233 ms vs 3,193 ms). Men also had slightly longer dwell time than women (3,281 ms vs 3,153 ms) during this task; see Table 1 and Fig. 3.

### AOI Analyses

#### Attractiveness

**Fig. 4:**
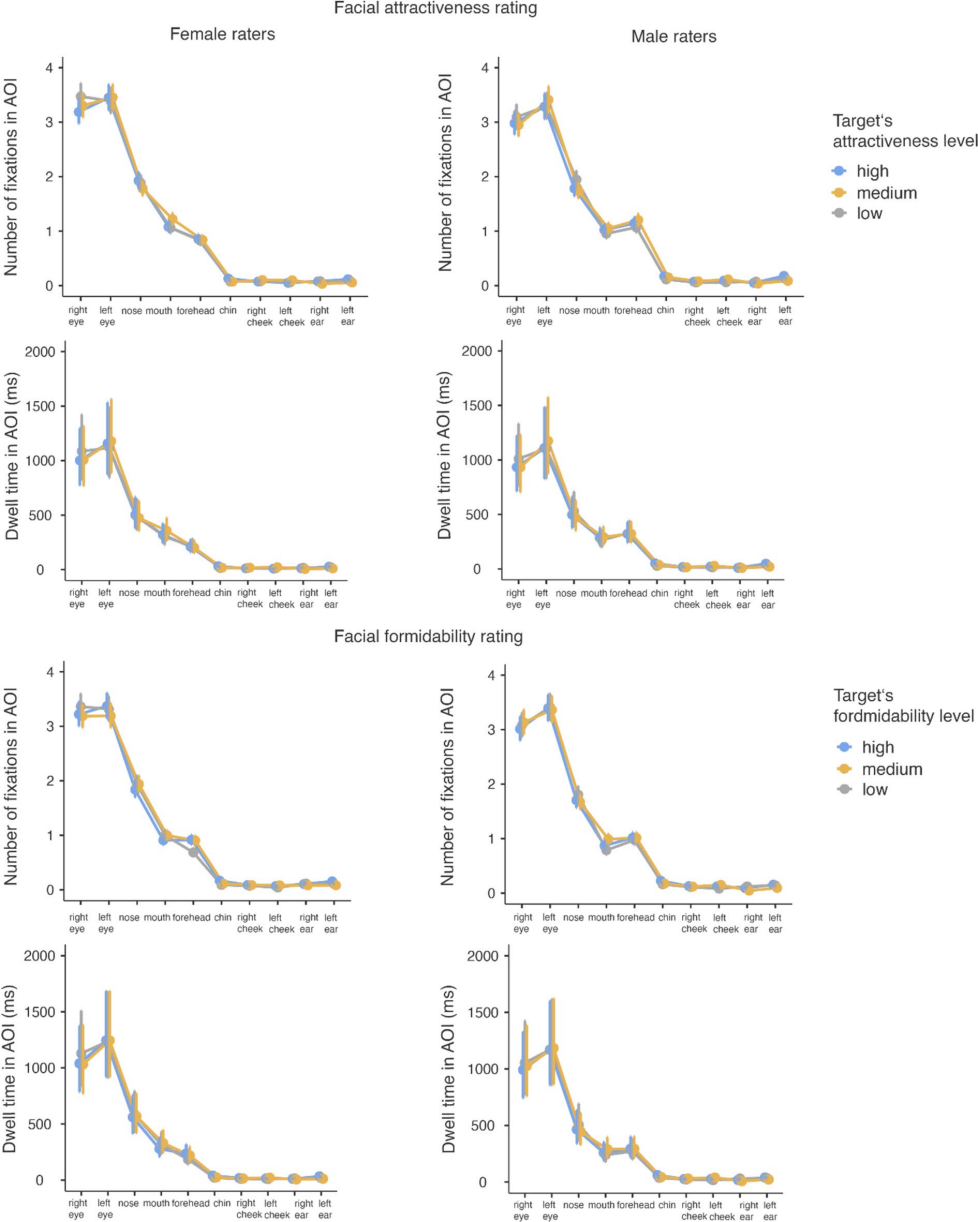
Differences in visual attention directed at individual AOIs in relation to the target’s level of attractiveness during a rating of facial attractiveness (top) and facial formidability (bottom). Female raters are in the left column, male raters in the right one. For each rating, the number of fixations on AOIs is in the top row and the dwell time in AOIs in the bottom row. Dots represent mean values, error bars show their 95% confidence intervals

A GLMM showed that the number of fixations in AOIs during the facial attractiveness rating task was statistically discernibly predicted by the AOI (*χ*^2^(9) = 20,483.46, *p* < 0.001) and by the target’s level of attractiveness (*χ*^2^(2) = 9.87, *p* = 0.007), but not by rater’s sex (*χ*^2^(1) = 3.74, *p* = 0.053). Interaction between AOIs and rater’s sex (*χ*^2^(9) = 140.83, *p* < 0.001) as well as interactions between AOIs and the target’s level of attractiveness (*χ*^2^(18) = 108.21, *p* < 0.001) were also statistically discernible. Interaction between rater’s sex and target’s level of attractiveness (*χ*^2^(2) = 1.70, *p* = 0.428) and between AOI, rater’s sex, and target’s level of attractiveness (*χ*^2^(18) = 17.08, *p* = 0.518) were, however, not statistically discernible. Men had on average statistically discernibly more fixations than women on the chin (0.144 vs 0.089 for men and women respectively), forehead (1.138 vs 0.839), and the left ear (0.123 vs 0.083). Regardless of their sex, participants made on average statistically discernibly more fixations on the chin of faces with high rather than low attractiveness level (0.149 vs 0.093) and on the left cheek of faces with a medium rather than low attractiveness level (0.107 vs 0.061). Further, regardless of sex, participants had on average statistically discernibly more fixations on the left ear of highly attractive as opposed to medium attractive faces (0.145 vs 0.071) and more fixations on the right ear of faces with a low rather than medium level of attractiveness (0.078 vs 0.035). For details, see Table 1 and Fig. 4.

In terms of the dwell time in AOIs during facial attractiveness rating, GLMM had shown that it is statistically discernibly predicted by the AOI (*χ*^2^(9) = 9,535.77, *p* < 0.001) and by target’s level of attractiveness (*χ*^2^(2) = 6.81, *p* = 0.033) but not by the rater’s sex (*χ*^2^(1) = 3.23, *p* = 0.072). There was also a statistically discernible interaction between the AOI and rater’s sex (*χ*^2^(9) = 58.46 *p* < 0.001) and between the AOI and target’s level of attractiveness (*χ*^2^(18) = 142.43, *p* < 0.001), but not between rater’s sex and target’s level of attractiveness (*χ*^2^(2) = 2.12, *p* = 0.346) or between the AOI, rater’s sex, and target’s level of attractiveness (*χ*^2^(18) = 23.84, *p* = 0.160). Men exhibited on average statistically discernibly longer dwell time on the chin and the left ear (37.8 ms and 30.8 ms, respectively) than women did (20.4 ms and 18.5 ms, respectively). Further, regardless of sex, participants exhibited a statistically discernibly longer dwell time on the chin of faces with a high rather than low attractiveness level (39.74 ms vs 21.04 ms) and on the left cheek of faces with a medium rather than high or low level of attractiveness (26.07 ms, 14.19 ms, 13.31 ms, respectively). Moreover, raters exhibited a longer dwell time on the left ear of highly attractive rather than medium attractive faces (35.90 ms vs 14.86 ms) and on the left ear of faces with a low as opposed to medium level of attractiveness (25.47 ms vs 14.86 ms). Finally, raters had a longer dwell time on the right ear of faces with a low as opposed to medium level of attractiveness (15.92 ms vs 6.39 ms) and on the right ear of faces with a high rather than medium level of attractiveness (11.11 ms vs 6.39 ms). For details, see Table 1 and Fig. 4.

#### Formidability

A GLMM showed that the number of fixations in AOIs during facial formidability rating was statistically discernibly predicted by the AOI (*χ*^2^(9) = 21,353.83, *p* < 0.001), target’s formidability level (*χ*^2^(2) = 21.18, *p* < 0.001), rater’s sex (*χ*^2^(1) = 11.58, *p* < 0.001), by interaction between the AOI and target’s level of formidability (*χ*^2^(18) = 109.03, *p* < 0.001), and by interaction between the AOI and rater’s sex (*χ*^2^(9) = 118.09, *p* < 0.001). It was not, however, predicted by interaction between the target’s level of formidability and rater’s sex (*χ*^2^(2) = 3.84, *p* = 0.147) or by interaction between the AOI, target’s level of formidability, and rater’s sex (*χ*^2^(18) = 27.52, *p* = 0.070). Men made on average statistically discernibly more fixations on the chin than women (0.186 vs 0.118 for men and women respectively), more fixations on the forehead (1.002 vs 0.828), more fixations on the left cheek (0.116 vs 0.062), and more fixations on the right cheek (0.120 vs 0.083). Regardless of sex, participants made statistically discernibly more fixations on the chin of faces with a high as opposed to low level of formidability (0.194 vs 0.120) and on the left cheek of faces with a medium as opposed to low level of formidability (0.112 vs 0.058). Moreover, they made more fixation on the left ear of faces with a high as opposed to medium level of formidability (0.155 vs 0.088) and more fixations on the right ear of faces with a low as opposed to medium level of formidability (0.119 vs 0.060). For details, see Table 1 and Fig. 4.

The GLMM showed that the *dwell time* on AOIs during facial formidability rating was statistically discernibly predicted by the AOI (*χ*^2^(9) = 7,592.15, *p* < 0.001), the target’s level of formidability (*χ*^2^(2) = 15.14, *p* < 0.001), rater’s sex (*χ*^2^(1) = 4.45 *p* = 0.035), by interaction between the AOI and target’s level of formidability (*χ*^2^(18) = 156.64, *p* < 0.001), and by interaction between the AOI and the rater’s sex (*χ*^2^(9) = 71.29, *p* < 0.001). It was not, however, predicted by interaction between the target’s level of formidability and rater’s sex (*χ*^2^(2) = 2.07, *p* = 0.355) or by interaction between the AOI, target’s level of formidability, and rater’s sex (*χ*^2^(18) = 8.19, *p* = 0.976). Men exhibited, on average, statistically discernibly longer dwell time on the chin (45.87 ms vs 27.26 for men and women, respectively), the left cheek (26.17 ms vs 14.14 ms), and the right cheek (26.24 ms vs 13.72 ms). Further, regardless of sex, participants had a statistically discernibly longer dwell time on the chin of faces with a high as opposed to low level of formidability (48.70 ms vs 27.81 ms), on the left cheek of faces with a medium as opposed to low level of formidability (29.12 ms vs 12.56 ms), on the left ear of faces with a high as opposed to medium level of formidability (35.99 ms vs 15.71 ms), on the right ear of faces with a high as opposed to medium level of formidability (14.42 vs 6.67 ms), and on the right ear of faces with a low as opposed to medium level of formidability (21.09 vs 6.67 ms). For details, see Table 1 and Fig. 4.

## Discussion

The main aim of the present study was to investigate men’s and women’s visual attention (measured as the number of fixations and dwell time) directed at real-life-sized photographs of male faces during attractiveness and formidability ratings. We have also explored whether and how the target’s level of attractiveness and formidability (low, medium, high) affects raters’ visual attention during each of the two kinds of judgments. In addition to the eyes, the nose, and the mouth, i.e., areas typically used in eye-tracking studies, we have defined a broad array of AOIs proposed by various morphometric studies. We found that during both types of judgments, men looked longer at the faces than women did. Raters of both sexes also looked longer at faces with a medium rather than high formidability level. Irrespective of the characteristics rated, both sexes paid the most visual attention to the eyes, nose, mouth, and forehead. Aside from that, we found statistically discernible variations also for other facial regions, such as the chin, the cheeks, and the ears.

### Whole Face Analyses

Contrary to our predictions, analyses of visual attention to the whole face showed that during attractiveness ratings, there was no association between the number of fixations on the face, target’s level of attractiveness, rater’s sex, or their interaction. Nevertheless, men showed a statistically discernibly longer dwell time during attractiveness rating than women did. For the formidability rating, again contrary to our expectations, we found no statistically discernible relationships for the number of fixations on whole faces. On the other hand, the dwell time on the whole face during formidability rating was predicted by the rater’s sex (men had marginally longer dwell times on faces than women did) and by the target’s formidability level (raters looked longer on faces with a medium rather than high level of facial formidability). These patterns do not follow our prediction according to which visual attention would increase with the target’s higher level of attractiveness or formidability.

The absence of a relationship between the number of fixations on the whole face, target’s attractiveness level, and rater’s sex is somewhat surprising because previous research reported that attractive faces are looked at more often and longer (Leder et al., 2016; Mitrovic et al., 2018). Even so, some studies (Leder et al., 2016; Mitrovic et al., 2018) reported merely a rather weak association between visual attention and attractiveness of male faces, and in the study by Mitrovic et al. (2016), it was not observed at all. Our results thus do have a precedent.

Why women’s visual attention to male faces did not differ in relation to the level of perceived attractiveness is more puzzling. Possible explanation might be that previous studies which demonstrated this association (e.g., Leder et al., 2016; Mitrovic et al., 2016, 2018) used different designs than our study. For instance, they used photographs with two people in one picture, with natural-looking settings and used a free-viewing paradigm—such setups may have facilitated a comparison between the stimuli faces and allow for the more attractive stimulus to capture rater’s visual attention more (Třebický et al., 2024b). Moreover, it is also possible that in our stimuli, the differences in attractiveness were not pronounced enough to generate a significant effect on visual attention, although differences between the mean ratings in individual categories do seem similar to previous studies (at least with respect to the high and low categories) which were able to demonstrate the effect (Kwart et al., 2012; Mitrovic et al., 2016).

Interestingly, we found that men had a marginally longer dwell time (Δ ~ 130 ms) on male faces during facial attractiveness ratings than women did. This may indicate the task difficulty. Men may need more time to extract the relevant information in such contexts (Jacob & Karn, 2003), especially if they are not used to judging other men’s attractiveness. Moreover, Mitrovic et al. (2016) suggested intrasexual comparison as an explanation of why men spend more time looking at male faces regardless of differences in facial attractiveness levels. This might also apply to our case: men may have been comparing themselves to the stimuli, thus needing more time for the assessment, while women did not. Along similar lines, that is, based on intrasexual comparison, we might explain the marginally longer dwell time of male participants during formidability judgments (Δ ~ 128 ms). In other words, we can speculate that in our male raters, the assessment of both male facial attractiveness and formidability may have been associated with a comparison between themselves and the target, and this translated to a longer gaze. In future studies, the use of both male and female stimuli may help resolve this issue. Further, raters looked longer at faces with a medium (as opposed to high) level of formidability, which may be the result of the ambiguity of these faces and raters’ uncertainty regarding their rating. This may have led to the prolonged gaze duration (Martín-Loeches et al., 2014). In any case, differences in dwell duration were rather small (Δ ~ 40 ms), and one could argue about their practical significance.

### Areas of Interest Analyses

Analyses which explored visual attention directed at individual AOIs showed that for attractiveness rating, the highest number of fixations and the longest dwell time were, as expected, directed at the eyes, nose, mouth, and also forehead (in our case including the top of a head and hair) (Table 1 and Fig. 4). The remaining areas received comparatively little direct visual attention. An exploration of interactions showed that men made marginally more fixations on the chin, forehead, and the left ear and had a longer dwell time on the chin and left ear than women did. Further, raters, irrespective of sex, made marginally more fixations and had a longer dwell time on the chin of faces with a high as opposed to low level of attractiveness and on the left cheek of faces with a medium as opposed to low level of attractiveness. Longer dwell times have also been found for the left cheek of faces with a medium as opposed to high attractiveness levels. Finally, we have observed some variation in the number of fixations and dwell time on the left and right ear in relation to the target’s level of attractiveness. But given that these differences were only in the order of fractions of fixations and tens of milliseconds, it would be farfetched to argue about their practical implications.

Analyses that investigated visual attention directed at particular AOIs showed that for formidability rating, the highest number of fixations and the longest dwell time were, as expected, likewise directed at the eyes, the nose, the mouth, and the forehead. Exploration of interactions had shown that men had statistically discernibly more fixations and longer dwell time on the chin and both cheeks and more fixations on the forehead than women did. Raters made marginally more fixations and had a longer dwell time on the chin of faces with a high as opposed to low level of formidability and on the left cheek of faces with a medium as opposed to a low level of formidability. In parallel to the attractiveness ratings, we found a variation for the number of fixations and dwell time on the right and the left ear in relation to the target’s level of formidability. As in the case of interactions within attractiveness ratings, the observed differences were small, and one might once again dispute their practical relevance.

Overall, the observed patterns of visual attention align with previous eye-tracking studies: they show that the eyes, the nose, and the mouth regions receive the most visual attention regardless of the context (i.e., characteristics being rated) (Chelnokova & Laeng, 2011; Hermens et al., 2018; Kwart et al., 2012). Some studies have also reported the forehead as an area of increased visual interest (Nguyen et al., 2009). We have likewise observed heightened visual attention toward the forehead in our study, especially in male raters. Windhager et al. (2011) showed forehead shape changes in relation to an individual’s strength, but otherwise, the forehead does not seem to be commonly connected with perceived formidability. It should be noted that while in our study, the AOI was classified as the forehead, it included hair, which may have attracted raters’ visual attention on its own. The heightened visual attention toward the eyes, the nose, and the mouth in our study is not surprising as these features are most conspicuous. However, it might not be the only reason why they attract attention; they also help in the assessment of intentions via facial expressions, following the direction of the target’s gaze or vocalization. This is important for first impression formation and was crucial for avoiding costly judgment errors in our evolutionary past (Kleisner & Saribay, 2019). However, this finding contrasts with some perception studies and with the findings of morphometric studies, which showed that besides the eyes, nose and mouth, the chin and the cheeks may also be important for judgments of attractiveness and formidability (Cunningham et al., 1990; Třebický et al., 2013; Windhager et al., 2011).

There are several possible reasons why the chin and the cheeks attracted only limited visual attention. Morphometric studies show that while the shape of certain (and multiple) facial regions may be associated with the rating of particular characteristics, their perceptual importance can vary. As outlined in the introduction, morphometric studies may detect shape deformation in multiple regions, but only some might be salient to the judgment. Another reason may have to do with certain limitations of eye-tracking in terms of indicating what raters actually see. Although we used life-sized stimuli (to allow for a more realistic visual search, as opposed to most previous eye-tracking research), which is a strong point of our study, the area covered by central vision may have covered multiple AOIs at once (see Fig. 2). As a result, although direct visual attention was not directed specifically at some facial features, they may have been in the rater’s field of central vision. It means that raters may have seen them and processed them without the eye-tracker detecting a direct fixation. This is well possible: It has been demonstrated that attractive faces which are even outside a person’s foveal vision capture visual attention (Sui & Liu, 2009), and another study has shown that when it comes to identification of attractive faces, there is no difference in the performance between the foveal and parafoveal vision (Guo et al., 2011). On top of that, people can detect attractive faces even using their peripheral vision (Guo et al., 2011).

Nevertheless, men in our sample had slightly more fixations and longer dwell time on the chin, more fixations on the forehead during both formidability and attractiveness ratings, and more fixations on cheeks during formidability judgments than women did. We suggest that these areas might contain information which helps men make the relevant judgments and that these regions are more important to men than they are to women. Finally, some of the variation in visual attention to the ears, which we explored, might be explained by certain specific characteristics of some of our targets. Being MMA fighters, some targets had “cauliflower” ears, which may have attracted some attention, as it might be unexpected to see but also possibly cue formidability. Still, it should be stressed that although we offer interpretations for the observed differences, they are quite small and one ought to be cautious regarding their importance.

Some previous studies have shown that the noses and mouths of attractive faces are fixated upon more often (Kwart et al., 2012). Our present findings do not bear this out. Instead, we found that the chin of highly formidable and highly attractive faces received more fixations and was looked at longer than these areas in faces with low attractiveness/formidability, and this was also the case for the left cheek of faces with the medium rather than high level of attractiveness/formidability. This might indicate a level of salience of these regions for faces with certain levels of formidability/attractiveness and the relevant judgments. It is also possible that irrespective of the type of judgment, one inspects the eyes, the nose, and the mouth, and in case of uncertainty about the judgment, one inspects further facial features, such as the cheeks and the chin, which then contribute to reaching a decision. Moreover, given that the patterns of visual attention were similar for judgments of both attractiveness and formidability and their respective levels, one could speculate that these two specific judgments involve similar visual attention.

## Limitations

Our study had certain limitations. We focused on young adults, persons at the stage of life when majority of mate choice takes place. Future studies should also investigate adolescents and seniors to gain insight into the developmental trajectories of visual attention to faces. Another possible limitation might be that our targets were MMA fighters. As such, they constituted a specific sample whose characteristics (e.g., broken noses, cauliflower ears) differed from the general population. The sample may have also suffered from a skewed attractiveness and formidability distribution or insufficient variation in their levels: The interindividual differences may have been too small to translate into measurable differences in visual attention. Moreover, our stimuli presentation design was not fully randomized in the traditional way. The presentation of stimuli in blocks (due to the study being part of a larger project) may have played a role. For instance, a stimuli block contained pictures of the same salience, which could have led to raters’ desensitization to the level of stimuli’s attractiveness/formidability.

## Conclusions

We have assessed visual attention to male faces and their particular features in the context of attractiveness and formidability judgments made by both men and women, whereby visual attention was measured both as the number of fixations and the dwell time. We observed that during both facial attractiveness and formidability judgments, men looked longer at faces than women did. Further, we found no effect of raters’ attractiveness and formidability level on their visual attention except that raters, irrespective of their sex, looked longer at faces with a medium as opposed to high level of formidability. Moreover, regardless of the rated characteristics, men and women directed the most visual attention to the eyes, nose, mouth, and forehead of the stimuli. Other facial regions received relatively little visual attention. We have detected small variations in visual attention directed at, e.g., the forehead, chin, cheeks, and the ears in relation to the rater’s sex and target’s level of attractiveness, and while we propose some interpretations, we are aware that these differences, while statistically discernible, need not be large enough to have any practical importance. To conclude, the eyes, the nose, and the mouth seem to be central to the evolution of facial perception as the most salient regions for gathering information about others. Future research should strive to further connect eye-tracking and GMM methods and investigate whether areas of important morphological deformations correspond to areas of increased visual attention.

## Data Availability

The data and Supplementary Materials associated with this research are available at https://osf.io/q6jt7/.
